# Systematic discovery of retina-enriched Rik genes identifies 1190005I06Rik as a novel modulator of visual signalling

**DOI:** 10.1186/s12967-026-07769-z

**Published:** 2026-01-30

**Authors:** Yu-Tong Liu, Qing Li, Xinghai Yu, Zi-Wu Wang, Jun-Yan Kang

**Affiliations:** 1 State Key Laboratory of Eye Health, Department of Ophthalmology, Shanghai Ninth People’s Hospital, Shanghai Jiao Tong University School of Medicine, Shanghai, China; 2 Institute of Ophthalmology and Visual Science, Shanghai Jiao Tong University School of Medicine, Shanghai, China; 3 Shanghai Key Laboratory of Orbital Diseases and Ocular Oncology, Shanghai, China; 4 Department of Histoembryology, Genetics and Developmental Biology, Shanghai Key Laboratory of Reproductive Medicine, Shanghai Jiao Tong University School of Medicine, Shanghai, China; 5 Shanghai Key Laboratory of Gene Editing and Cell Therapy for Rare Diseases, Fudan University, Shanghai, China

**Keywords:** Retina, Visual function, 1190005I06Rik, Multi-omics

## Abstract

**Background:**

High‑throughput transcriptome projects have revealed thousands of mammalian genes with little or no functional annotation. Among these are hundreds of loci assigned provisional “Rik” identifiers following discovery in the RIKEN cDNA annotation effort. Although often dismissed as genomic dark matter, such genes may encode tissue‑restricted proteins that modulate physiologic functions and influence disease. The retina is a highly specialised neural tissue and a common site of inherited disorders; understanding its molecular repertoire could illuminate novel therapeutic avenues.

**Methods:**

We integrated bulk RNA‑seq from ten adult mouse tissues, evolutionary and domain analysis, single‑cell RNA‑seq, and CRISPR/Cas9 gene disruption to systematically catalogue protein‑coding Rik genes enriched in the retina and test the function of a representative gene.

**Results:**

A rigorous differential expression analysis identified 44 Rik genes with robust retina‑specific expression compared with nine non‑retinal tissues. Many of these genes lack orthologues beyond rodents, while others show broad conservation, illustrating a continuum from lineage‑restricted to conserved retinopathy candidates. Single‑cell transcriptomics revealed that these genes are expressed across retinal cell types, with the highest aggregate expression in cone photoreceptors and inner interneurons. To evaluate physiological significance, we generated a *1190005I06Rik* knockout mouse. Although retinal architecture appeared normal, loss of *1190005I06Rik* enhanced electroretinogram b‑wave amplitudes and altered light‑avoidance behaviour, indicating that this previously uncharacterised gene acts as a negative modulator of visual signalling.

**Conclusions:**

We present a curated atlas of retina‑enriched Rik genes and demonstrate that 1190005I06RIK modulates retinal circuit function. This resource expands the molecular landscape of the retina and provides new candidates for the genetic basis of inherited retinal disease. Our findings underscore that unannotated genes may exert measurable effects on sensory processing and warrant systematic exploration in the context of human ocular disorders.

**Supplementary Information:**

The online version contains supplementary material available at 10.1186/s12967-026-07769-z.

## Introduction

Advances in genome sequencing and annotation have greatly expanded the catalogue of mammalian genes. Projects such as the RIKEN Mouse Gene Encyclopedia have reported thousands of previously unrecognised transcripts, many of which were provisionally named using “Rik” identifiers [1, 2]. These loci are part of the so‑called genomic “dark matter”: they lack functional annotation, often show restricted expression, and are absent from curated pathway databases. Across eukaryotic genomes roughly 10–20% of genes fall into this category [3, 4], and their fast evolutionary turnover suggests that some may encode lineage‑specific innovations [5–7]. Rather than being transcriptional noise, uncharacterised genes provide an opportunity to discover novel regulators of tissue physiology and disease [6, 8].

The retina is a complex sensory tissue that converts light into neural signals and is susceptible to over 280 monogenic disorders [9–11]. Many of the > 60 phototransduction and structural genes that underlie human inherited retinal diseases (IRDs) were discovered by virtue of their high expression in retina relative to other tissues [12]. With the advent of high‑throughput sequencing and single‑cell transcriptomics, comprehensive retinal gene catalogues have been generated [13–15]. These studies have delineated major cell types and identified cell‑type markers, yet uncharacterised genes were largely overlooked as attention focused on known components. Considering that orphan genes often adopt tissue‑specific functions [3, 4], we hypothesised that a subset of Rik genes might be highly enriched in the retina and contribute to visual function or disease susceptibility.

Here we systematically interrogated the mouse transcriptome to identify and characterise Rik genes with retina‑specific expression. By integrating bulk RNA‑seq from ten adult tissues, we generated a comprehensive expression map and applied stringent criteria to define a set of retina‑enriched protein‑coding Rik genes. We then used comparative genomics, domain analysis and single‑cell RNA‑seq to evaluate conservation, structural features and cellular context. Finally, we employed CRISPR/Cas9 to disrupt a representative gene and assess its impact on visual physiology and behaviour. Through this multi‑pronged approach we sought to illuminate new molecular players in the retina and lay a foundation for translational investigations into IRDs.

## Results

### Transcriptomic identification of retina-enriched Rik genes

**Fig. 1:**
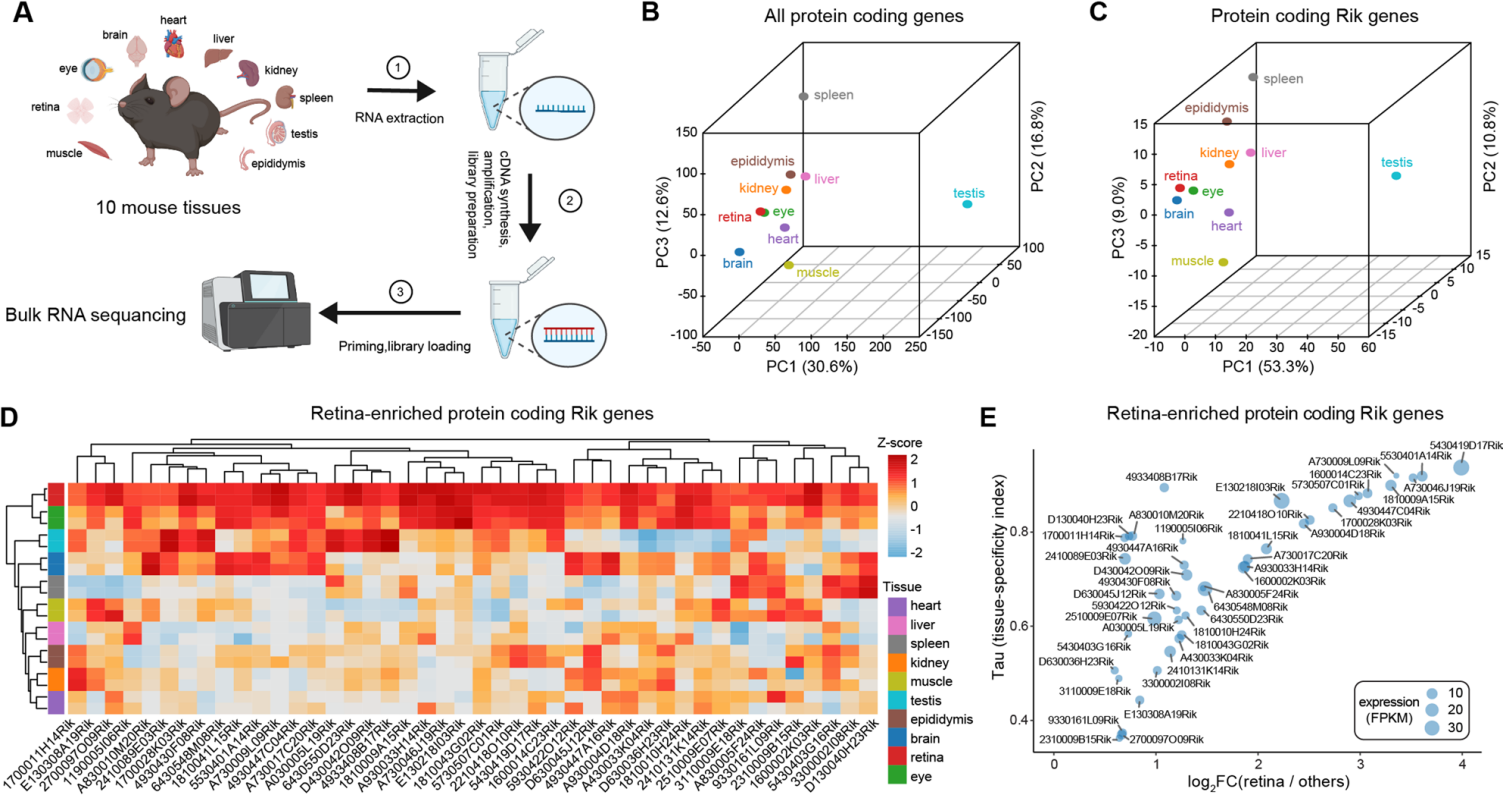
Transcriptomic identification of retina-enriched Rik genes in adult mouse. (**A**) Schematic of RNA-seq experimental and analytical pipeline. (**B** and **C**) Principal component analysis (PCA) based on global expression of all protein-coding genes and Rik genes. PCA plot using mean FPKM values of all protein-coding genes (**B**) and Rik genes (**C**) across 10 mouse tissues. (**D**) Heatmap of retina-enriched Rik gene expression across tissues. 44 Rik genes were defined as retina-enriched using the following criteria: log_2_FC (retina vs. other tissues) > 0.58, adjusted *p*-value < 0.05 (DESeq2), and mean retina FPKM > 0.1. Z-transformed log₂FPKM values were used for hierarchical clustering across tissues. (**E**) Scatter plot with log_2_FC (retina vs. other tissues) on the x-axis and tissue specificity index τ on the y-axis. Circle size represents mean retina FPKM

To establish a resource of uncharacterised genes relevant to retinal biology, we performed bulk RNA-seq of ten adult mouse tissues (Fig. 1A). Principal component analysis of global transcriptomes showed clear separation of tissues and clustered retina closest to eye (Fig. 1B). Restricting the analysis to Rik genes accentuated similarities among neural tissues, with retina, eye and brain forming a cluster distinct from non‑neural organs (Fig. 1C). Clustering the top 200 protein-coding genes revealed tissue-specific expression modules (Figs. S1A–S1B). As expected, the retina-enriched genes showed enrichment of Gene Ontology (GO) terms related to visual perception, energy production, and phototransduction (Fig. S1C). These results demonstrate that our transcriptomic dataset faithfully recapitulates known tissue specializations. Notably, we observed a similar pattern when focusing on Rik genes: these also formed tissue-specific clusters (Fig. S1D), suggesting that Rik genes are also expressed in a tissue-selective manner, analogous to canonical genes.

Focusing on the retina, we identified 44 protein‑coding Rik genes that were robustly and selectively expressed in the retina (Fig. 1D). The vast majority also exhibited high tissue specificity indices (τ) and large effect sizes for differential expression (Fig. 1E), confirming that these Rik genes are both strongly upregulated in retina and largely restricted to it. Supporting this, a retina-enrichment score summarising expression of the 44 genes across tissues showed a pronounced peak in the retina compared with any other tissue (Fig. S1E), highlighting their tissue specificity.

This set of genes, hereafter referred to as retina‑enriched Riks, represents an unappreciated cohort of protein‑coding loci potentially relevant to retinal physiology.

### Evolutionary conservation and protein features of retina-enriched Rik genes

**Fig. 2:**
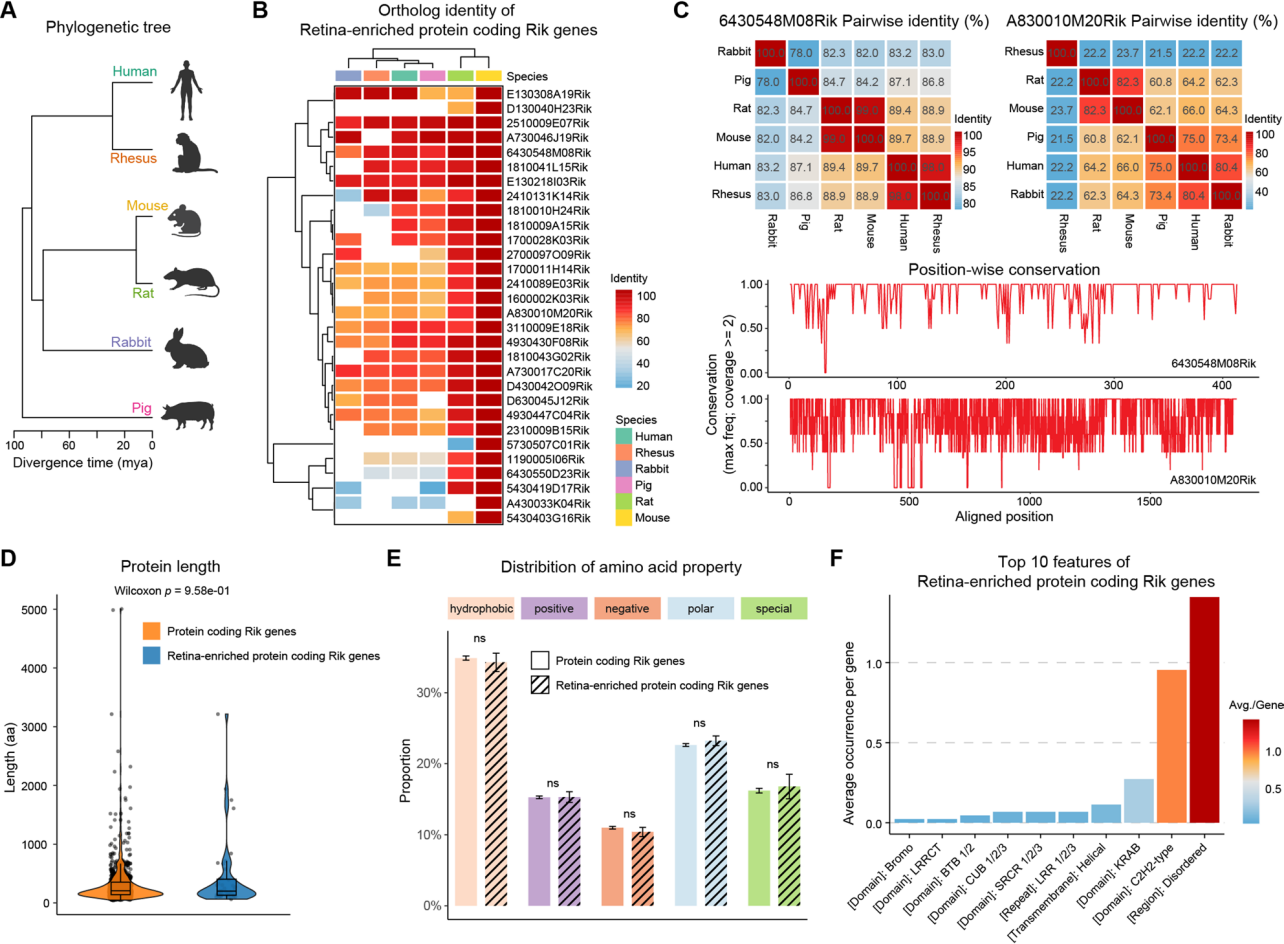
Evolutionary conservation and protein properties of retina-enriched Rik genes. (**A**) Phylogenetic relationship among representative mammalian species. (**B**) Heatmap of pairwise amino acid identity (percentage) for orthologous proteins aligned to mouse retina-enriched Rik sequences. Rows represent genes; columns indicate target species. White indicates undetected orthologs based on Ensembl orthology data. (**C**) Conservation plots include pairwise identity matrices and position-wise conservation scores for two cases. (**D**) Violin plot comparing amino acid sequence lengths. Retina-enriched Rik proteins are not significantly different (Wilcoxon test *p* > 0.01). (**E**) Bar chart displaying the relative abundance of five physicochemical amino acid classes (hydrophobic, polar, positive, negative, special: proline/glycine). Proteins were grouped by retina-enriched versus other Rik genes. (**F**) Top 10 annotated structural features retrieved from UniProt (Swiss-Prot and TrEMBL entries), ranked by average occurrence per protein

We next assessed the evolutionary conservation of the retina-enriched Riks. Orthologues were sought across five non‑mouse mammals (rat, rabbit, pig, macaque and human) spanning approximately 90 million years of divergence (Fig. 2A). Only about 70% (31/44) of the retina-enriched Riks had orthologs in at least one other species, whereas the remaining ~ 30% appeared to be restricted to mouse or rodents (Figs. S2A–S2B). Even among those with orthologs, conservation ranged from nearly complete identity between mouse and rat to less than 25% amino‑acid identity between rodents and primates (Figs. 2B–C). Notably, 24 of the 44 retina-enriched Riks have human orthologues, and these orthologues retain retinal-enriched expression to varying degrees in human transcriptomic datasets (Fig. S2C). Thus these genes comprise a continuum from deeply conserved to rodent‑specific. Such lineage‑restricted genes are plausible candidates for species‑specific visual adaptations, whereas conserved members may perform core retinal functions common to mammals.

To explore possible functions, we compared the proteins encoded by retina‑enriched Riks with those of other Rik genes. Protein length distributions and amino‑acid composition were similar between groups (Figs. 2D–E), alleviating concerns that they might all be tiny peptides or outliers in primary structure. However, domain analysis revealed striking differences: retina‑enriched Riks were enriched for tandem C2H2 zinc fingers and KRAB repression domains, suggesting transcriptional regulatory roles; they also contained scavenger receptor cysteine‑rich (SRCR), CUB, leucine‑rich repeat (LRR and LRRCT), BTB/POZ and bromodomains at higher frequency than non‑retinal Riks (Figs. 2F, S2D–S2E). Many of these domains mediate protein–protein interactions, extracellular matrix binding or chromatin engagement. Consistent with these annotations, AlphaFold2 structural predictions showed arrays of zinc fingers, globular LRR or CUB modules and extensive intrinsically disordered regions (Fig. S3). Taken together, the domain profile suggests that retina‑enriched Riks may function as transcriptional modulators or secreted/membrane proteins involved in cellular communication within the retina.

Overall, these results revealed that the retina-enriched Rik proteins have a distinct feature profile, and these unique structural characteristics support the idea that these Rik proteins may perform specialized functions not covered by canonical retinal proteins.

### Single-cell resolved dynamics of retina-enriched Rik gene expression during retinal maturation

**Fig. 3:**
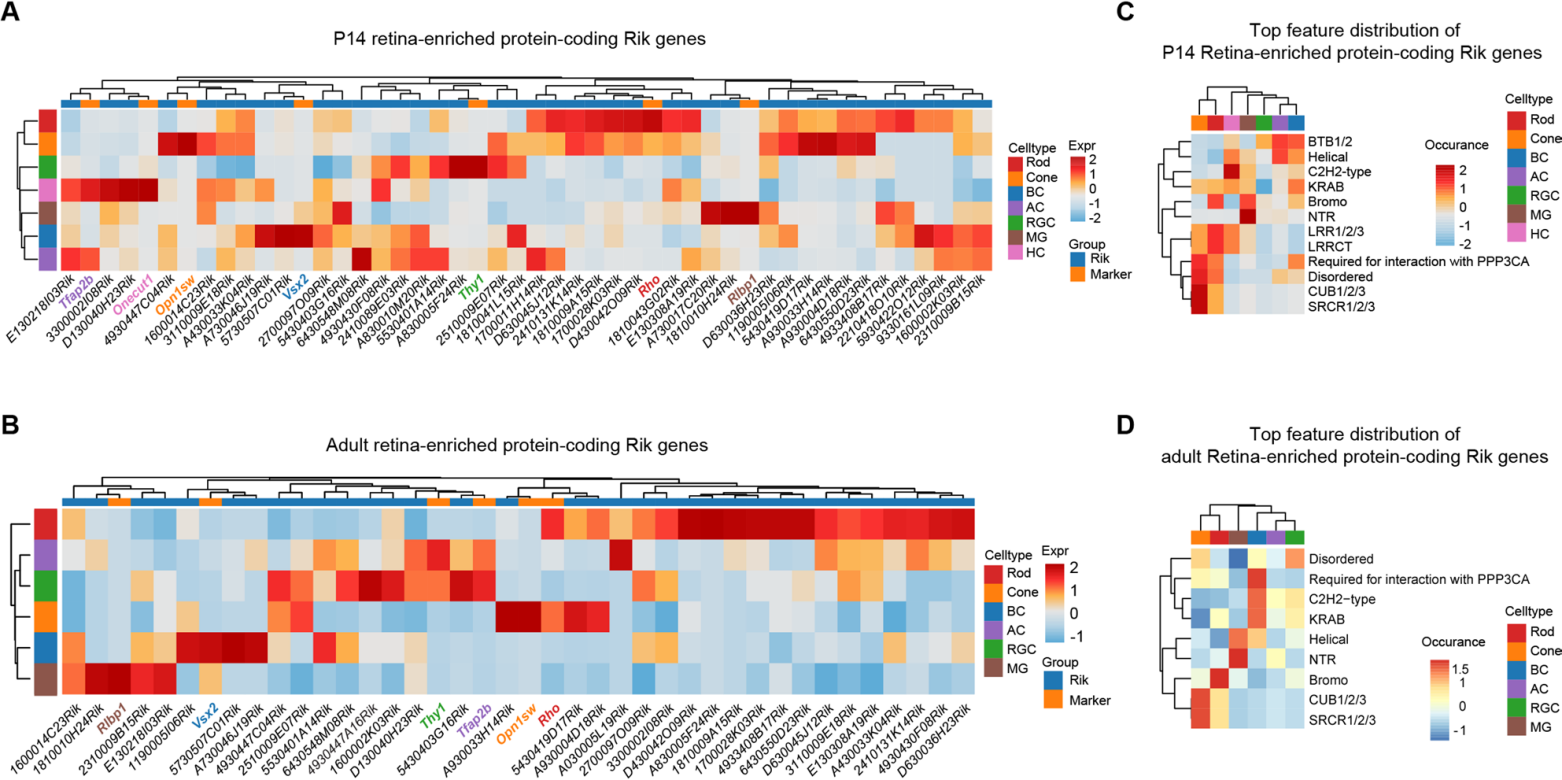
Single-cell characterization of retina-enriched Rik genes in the mouse retina. (**A–B**) Heatmap depicting the average expression of retina-enriched Rik genes alongside key canonical marker genes across major cell types in P14 (**A**) and adult (**B**) retinas. Each column corresponds to a gene, and each row to a cell type. Expression values are log-normalized and Z-scored by row. (**C–D**) Heatmap showing the relative feature–cell type enrichment of retina-enriched RIK proteins in P14 (**C**) and adult (**D**). For each structural protein feature (e.g., SRCR, Disordered, Helical, LRR, etc.), the average expression across cell types was first computed and then normalized per gene (i.e., row-wise Z-score)

To place the retina‑enriched Riks in a cellular context, we interrogated two single‑cell RNA‑seq atlases of the mouse retina at postnatal day 14 (P14) and at an adult stage (3 months of age; 3M) [15, 16], resolving major retinal cell types according to established marker gene expression (Figs. S4A–S4B). Notably, because of the limited sequencing depth of single-cell profiling, not all retina-enriched Riks were detected, and in the adult dataset we further excluded horizontal cells owing to insufficient cell numbers for robust analysis. Summation of expression across the retina-enriched Riks revealed that the cumulative signal was highest in cone photoreceptors and retinal interneurons at P14 (amacrine, bipolar and horizontal cells), whereas in adult retina it was concentrated in cones, bipolar cells and retinal ganglion cells, exhibiting adaptive shifts in these Rik genes deployment during retinal maturation (Fig. S4C). Indeed, quantification based on specificity score (where lower entropy indicates expression concentrated in fewer cell types) confirmed that gene rank order differed between the P14 and adult retinas (Fig. S4D). Unsupervised clustering of retina‑enriched Rik genes, alongside a set of canonical cell-type markers further revealed an overall increase in cell-type specificity in adult retina compared with P14; for example, *1190005I06Rik* showed high expression across cones, bipolar cells and rods at P14 but was predominantly enriched in bipolar cells in adult retina (Figs. 3A–B).

Beyond expression patterns, we asked whether the structural features enriched in the retina Rik proteins show bias toward certain cell types. Mapping protein features onto the single‑cell expression matrix revealed stage-dependent associations: at P14, photoreceptors predominantly expressed Rik genes bearing extracellular interaction domains (SRCR and CUB), amacrine cells preferentially expressed genes encoding BTB domains and helical coiled-coil regions, and Müller glia were enriched for Riks with NTR and bromodomains (Fig. 3C). In adult retina, SRCR- and CUB-containing Riks remained most prominent in cones, whereas bromodomain-containing Riks were biased toward rods and NTR- and helical-domain Riks were enriched in Müller glia (Fig. 3D). Notably, several feature categories exhibited convergence toward fewer cell types with maturation, mirroring the gene-level refinement observed above. For example, Riks with PPP3CA-interacting domain shifted from broader expression across rods, cones and bipolar cells at P14 to predominantly bipolar expression in adults, and KRAB-domain Riks (largely outside RGCs at P14) became more bipolar-enriched in adults. These patterns were not observed for the broader Rik gene family (Fig. S4E), consistent with feature–cell-type coupling that is preferentially associated with retina-enriched Riks.

In summary, these data provide a first glimpse into the potential cellular contexts for these formerly unannotated genes. Meanwhile, the enrichment of many Riks in restricted cell types raises the question of whether they contribute to cell type-specific functions.

### Functional assessment of a novel retinal gene: 1190005I06Rik

**Fig. 4:**
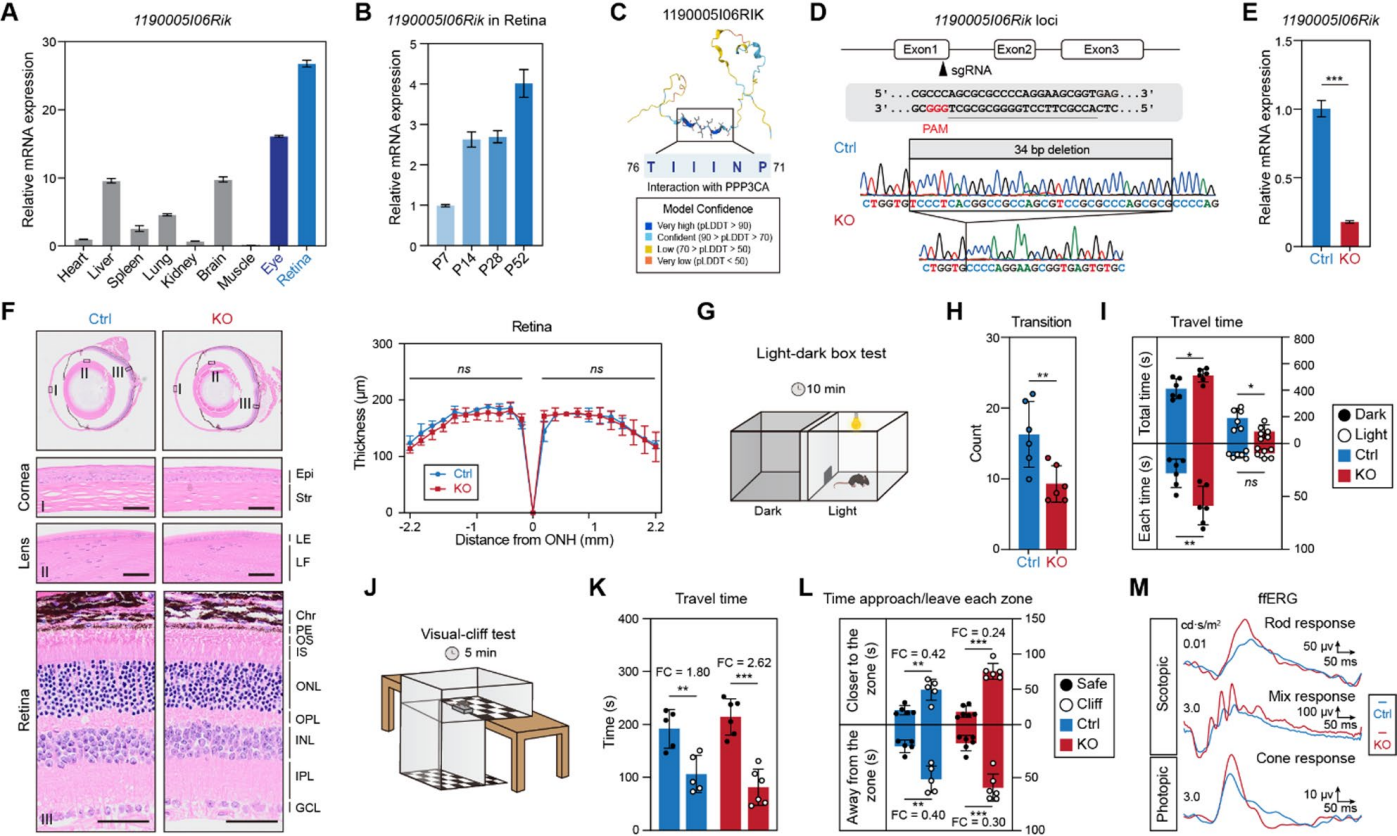
Functional assessment of *1190005I06Rik* knockout mice. (**A–B**) RT–qPCR analysis of *1190005I06Rik* in indicated mouse tissues (**A**) and mouse retina from P7 to P52 (**B**). Expression levels were normalized to β-actin. Data represent mean ± SEM (*n* = 3). (**C**) Schematic diagram of the alpha-fold predicted 1190005I06RIK protein, highlighting a PPP3CA-interacting domain. (**D**) Schematic diagram illustrating the CRISPR/Cas9 targeting site in the 1st exon of *1190005I06Rik*. A 34-bp deletion is confirmed by Sanger sequencing. (**E**) RT–qPCR analysis of *1190005I06Rik* in the retinas of control and KO mice. Data represent mean ± SEM (*n* = 3). **(F)** Left, representative H&E staining of paraffin ocular sections from adult control and KO mice. Right, spider graphs showing the thicknesses of control and KO retinas. Epi, corneal epithelium; Str, stroma; LE, lens epithelial cells; LF, lens fibre cells; Chr, chroid; PE, pigment epithelium; OS/IS, outer/inner segements; ONL, outer nuclear layer; OPL, outer plexiform layer; INL, inner nuclear layer; IPL, inner plexiform layer; GCL, ganglion cell layer; ONH, optic nerve head. Scale bar, 50 μm. Data represent mean ± SD (*n* = 3). **(G)** Diagram of the light–dark box test (*n* = 6). **(H)** Quantification of transitions between light and dark chambers. Bar plots represent mean ± SD. **(I)** Quantification of total time (top) and average duration (bottom) of visit spent in the dark (black) and light (white) zones for control and KO mice. (**J**) Diagram of the visual cliff test (*n* = 6). (**K**) Quantification of travel time in the safe (black) and cliff (white) zones for control and KO mice. **(L)** Quantification of average distance from the centre (top) and to the border (bottom) in each zone. **(M)** Representative waveforms of scotopic and photopic ERG in control and KO mice. Significant differences (Student’s *t*-test) are marked with asterisks: *p < 0.05, ** *p* < 0.01, *** *p* < 0.001

To test whether retina‑enriched Riks have physiological relevance, we selected *1190005I06Rik* for functional analysis, prioritizing candidates that retained retinal enrichment across species and offered a tractable mechanistic hypothesis. This gene was strongly enriched in the retina in both mouse and human (Figs. 1D, S2C), and its expression increased during postnatal retinal development (Figs. 4A–B). Notably, its single-cell expression pattern sharpened during retinal maturation, shifting from broader expression at P14 to predominant enrichment in bipolar cells in adult retina, indicating a potential role in supporting bipolar-cell function. In line with this notion, *1190005I06Rik* is predicted to encode a protein harbouring a domain that interacts with PPP3CA (Fig. 4C), the catalytic subunit of calcineurin, a key regulator of synaptic signalling [17, 18]. To directly test *1190005I06Rik*’s function, we generated a knockout (KO) mouse line using CRISPR/Cas9 (Fig. 4D). The engineered allele introduced a frameshift deletion, and germline transmission was confirmed by PCR genotyping (Figs. 4D, S5A). RT–qPCR analysis confirmed a marked loss of *1190005I06Rik* transcripts in KO retinas (Fig. 4E), validating the successful generation of a *1190005I06Rik* null mouse line. KO mice were viable, fertile, and had normal size and ocular morphology (Fig. S5B). Histological analysis of ocular sections revealed a well-preserved laminar organization of the cornea, lens, and retina in KO mice (Fig. 4F). Moreover, there were no measurable alterations in the thickness of the outer nuclear layer (ONL) or inner nuclear layer (INL) of the retina between KO mice and control littermates (Fig. S5C). Thus, *1190005I06Rik* is dispensable for gross retinal development.

To assess functional consequences of *1190005I06Rik* deletion, we firstly subjected control and KO mice to a light-dark box test [19] (Fig. 4G). KO mice exhibited significantly fewer transitions between the light and dark zones (Fig. 4H), suggesting reduced exploratory drive toward the illuminated area. In line with this, KO mice spent less time in the illuminated zone, more than doubling the difference in total occupancy between the two zones (Figs. 4I, S5D), implying heightened avoidance of brightness. Consistent with this interpretion, KO animals in the illuminated zone travelled shorter distances with straighter paths, as shown by a marked reduction of turn angle (Fig. S5E). By contrast, measures of spatial orientation (such as heading error and distance from zone centres or borders), rotational behaviour and centre‑area occupancy were indistinguishable from controls (data not shown). To probe other potential visual impairment, we carried out visual-cliff test [20] (Fig. 4J). KO animals displayed comparably to controls on core cliff measures, such as total time spent on shallow versus deep sides, entry/exit counts, and latencies, indicating preserved depth perception and basic risk evaluation (data not shown). However, KO mice displayed a stronger preference for the safe platform and maintained greater distances from the cliff edge (Figs. 4K–L), suggesting altered risk assessment under visually demanding conditions.

To test this mechanistic inference, we performed full-field electroretinography (ffERG). KO mice showed enhanced b-wave amplitudes (Fig. 4M), indicating an elevated gain of photoreceptor-bipolar signalling, which would be expected to alter the salience of light transitions and edge cues and thereby bias exploratory decisions toward darker or “safer” regions. This interpretation is consistent with the maturation-associated sharpening of *1190005I06Rik* expression toward bipolar cells and with the predicted PPP3CA-interacting domain in the encoded protein, raising the possibility that 1190005I06RIK constrains calcineurin-dependent synaptic signalling and/or excitability within the bipolar pathway. These considerations further support a model in which loss of *1190005I06Rik* primarily perturbs visual processing rather than emotional state, with the behavioural shifts representing downstream consequences of altered retinal circuit gain.

Together, these results suggest that *1190005I06Rik* plays a modulatory role in the retina, perhaps acting as a brake on photoreceptor or bipolar cell activity under normal conditions. Removing the gene lifts that brake, resulting in supranormal retinal responses. One possible mechanism is that *1190005I06Rik* could encode a protein that interacts with phototransduction or synaptic machinery to dampen signaling. Regardless, the key point is that a gene with no prior information has a measurable effect on visual system function when disrupted. This proof-of-concept finding validates our approach of targeting retina-enriched uncharacterized genes, and it motivates deeper studies on this and other genes from the set, including whether they might influence susceptibility to retinal stress or disease models.

## Discussion

Our integrated, multi‑level analysis of the mouse transcriptome uncovers a previously hidden landscape of retina‑enriched Rik genes and demonstrates that members of this “dark genome” contribute to visual circuit function. By combining bulk and single‑cell RNA sequencing, comparative genomics and functional genetics we build an atlas of 44 protein‑coding Rik genes that display robust retina specificity. Domain annotations reveal that these proteins are enriched for motifs that mediate DNA binding, chromatin regulation and intercellular signalling, suggesting that unannotated genes have the potential to modulate transcriptional programmes and extracellular communication in the retina. We further show that deletion of one representative gene, *1190005I06Rik*, enhances electroretinogram responses and alters exploratory behaviour, establishing a direct link between a previously uncharacterised gene and retinal physiology. Together, these findings argue that the genomic dark matter encodes modulators of specialised neural circuits.

From a translational perspective, our atlas expands the roster of candidate genes for inherited retinal diseases (IRDs). Despite major advances in identifying IRD genes, a substantial fraction of cases still lack a molecular diagnosis. Our retina‑enriched Rik list provides a curated set of genes whose high, selective expression in the retina makes them compelling candidates for unexplained IRDs [11]. Notably, some of the Riks we identify have human orthologues with conserved domains; others are rodent‑specific but may point to analogous pathways in primates. For instance, the human orthologue of *6430548M08Rik* (*KIAA0513*) has been implicated in neurological phenotypes [21, 22]. The demonstration that 1190005I06RIK may act as a brake on retinal signalling suggests that loss‑of‑function mutations in its human counterpart could contribute to hyperexcitability or neurodegeneration. Conversely, modulation of such negative regulators might offer therapeutic avenues to boost retinal responses in diseases characterised by photoreceptor loss or synaptic dysfunction.

Our evolutionary and structural analyses reveal a continuum from conserved to lineage‑restricted Riks. Some genes are retained across mammals, consistent with ancient roles in retinal biology, whereas nearly one‑third lack orthologues outside rodents, suggesting that the retina can co‑opt new genes to fine‑tune its function. Intriguingly, retina‑enriched Riks are enriched for C2H2 zinc fingers, KRAB repression modules, LRRs and scavenger receptor domains, features associated with gene regulation and ligand interactions. Single‑cell analysis shows that photoreceptors preferentially express Riks with extracellular interaction domains (SRCR and CUB), while inner neurons and glia favour genes with BTB or bromodomains, implying a division of labour across cell types. These domain–cell‑type correlations generate testable hypotheses: for example, Riks expressed in Müller glia with NTR/bromodomains may regulate chromatin states during gliosis, whereas those in cones with SRCR domains might participate in phagocytosis of shed outer segments.

Beyond the retina, our work illustrates how systematic mining of unannotated genes can illuminate organ‑specific biology. The retina serves as an ideal proof‑of‑concept because its major cell types and functional outputs are well characterised. Applying a similar framework to other organs and tissues could reveal novel regulators of immunity, metabolism or reproduction. The approach also underscores the importance of integrating computational predictions with in vivo experimentation: while sequence and expression patterns suggest potential roles, only functional perturbation revealed that 1190005I06RIK dampens retinal signalling. Exploring the remaining retina‑enriched Riks through loss‑ and gain‑of‑function studies, along with biochemical assays to map their interacting partners, will be essential to delineate the pathways they influence.

Several limitations should be considered. First, our transcriptomic analyses were conducted in adult tissues; some Riks may exhibit dynamic expression during development or ageing. Second, while our behavioural tests focused on light‑driven exploration, more refined visual assays could reveal subtler phenotypes. Third, although we highlight domain enrichments, definitive biochemical functions remain to be determined. Finally, translation to human disease will require verification that the human orthologues of retina‑enriched Riks are expressed in human retina and mutated in patients. Despite these caveats, our study provides a valuable resource and a template for interrogating genomic dark matter in a translational context.

## Materials and methods

### Mice

CRISPR/Cas9 genome editing was used to delete 34 bp in exon 1 of *1190005I06Rik*. A single guide RNA targeting ACCGCTTCCTGGGGCGCGCT was microinjected into fertilised zygotes together with Cas9 mRNA. Founder animals were screened by PCR using primers 5′‑TGCAGGCAGAGCAGGCCG‑3′ and 5′‑ATCTCCACGATCCTGGGGTCCC‑3′; the deletion allele produced a 34‑bp smaller product than the wild type. Heterozygous founders were bred to obtain homozygous knockout and wild‑type littermates for experiments.

### RNA-seq and comparative transcriptomics

Tissues were dissected from adult mice and snap‑frozen. All ten tissues were sequenced in the same batch with two biological replicates per tissue. Total RNA was extracted using column‑based kits. Libraries were prepared using the TruSeq RNA Sample Preparation kit (Illumina) and sequenced on a NovaSeq 6000 platform (Epiobitek, China). Reads were trimmed and aligned to the mouse mm10 genome using STAR. Gene counts were obtained and normalised with DESeq2 [23]. Genes were considered retina‑enriched when log_2_FC > 0.58 and adjusted *P* < 0.05 (Benjamini–Hochberg correction) and mean retina FPKM > 0.1. In this study, only Rik genes annotated as protein_coding in Ensembl were retained. Non-coding Rik genes, annotated as lncRNA, pseudogene, and other categories, were excluded.

GO-term enrichment analysis was performed using the R package clusterProfiler [24] with a Benjamini-Hochberg multiple testing adjustment under FDR cutoff of 0.05. Enriched GO-terms of biological process were plotted for visualization. All figures from computational analysis were made using the ggplot2 [25] and ComplexHeatmap [26] packages.

### scRNA-seq analysis

Single‑cell expression data were obtained from public scRNA‑seq datasets (GSE63472 and GSM7184519) [15, 16].

For P14 datasets, the raw gene expression matrix was processed using Seurat [27], starting with Log-Normalization (scale factor = 10,000) and the identification of 2,000 highly variable features via the vst method. After scaling the data, PCA was performed to retain 50 PCs, followed by batch effect correction using Harmony. For downstream clustering, an SNN graph was constructed using the first 20 Harmony dimensions (k = 20), and cell clusters were identified using the Louvain algorithm using resolutions 1.0. Finally, the dataset was visualized using UMAP based on the same 20 Harmony dimensions, employing a cosine metric and a min.dist of 0.3 to project cells into two-dimensional space.

For 3 M datasets, the analysis began with quality control, filtering cells to retain those with 200 to 6,000 detected features and less than 10% mitochondrial gene content. Data were then normalized and scaled using SCTransform, with mitochondrial percentage regressed out to minimize technical noise. Following PCA (30 PCs), a UMAP dimensionality reduction and an SNN graph were constructed using the top 20 dimensions. Finally, cell clustering was performed using the Louvain algorithm at a resolution of 0.3 to identify distinct cell populations.

Cell types were assigned based on established marker genes. For each retina‑enriched Rik gene, average expression and an entropy‑based specificity score were computed across cell types. Protein feature–cell-type enrichment was assessed by first calculating cell-type–averaged expression for genes carrying a given domain and then performing within-gene Z-scoring across cell types. Comparisons with the entire Rik gene set served as controls.

### Phylogenetic divergence times and orthologue assignment

Divergence times among the six mammals (*Homo sapiens*, *Macaca mulatta*, *Oryctolagus cuniculus*, *Sus scrofa*, *Rattus norvegicus and Mus musculus*) were obtained from the TimeTree database (http://www.timetree.org). Species names were submitted to the TimeTree web interface (Timetree; default settings), and the resulting dated phylogeny was exported. The corresponding Newick string with branch lengths (in million years ago, MYA) was used for downstream plotting in R (ape::read.tree). Tip labels were standardised to species names, and the phylogram was rendered with branch lengths preserved (ape::plot.phylo; use.edge.length = TRUE).

Orthologues were identified using Ensembl Compara (release 109) and retrieved via BioMart by extracting, for each mouse Ensembl gene ID, the homolog Ensembl gene ID, orthology type and the Compara-reported percent identity. Orthologues were considered present when a valid homolog gene ID was returned; if multiple mappings existed for a given gene–species pair, the entry with the highest percent identity was retained. Conservation breadth was quantified as the number of non-mouse species with a detected orthologue. For representative case studies, mapped Ensembl gene IDs were linked to UniProt accessions (preferring Swiss-Prot when available) to retrieve protein FASTA sequences, which were aligned using MUSCLE (with Clustal Omega as a fallback). Pairwise amino-acid identity was calculated from the alignment as the fraction of identical residues across positions without gaps, and column-wise conservation was summarized as the maximum residue frequency among non-gap amino acids.

### RT-qPCR analysis

Total RNA from retinas or other tissues was extracted with TRIzol (Invitrogen). First‑strand cDNA synthesis was carried out using a Reverse Transcription Kit with oligo(dT) and random primers. qPCR was performed using SYBR Green Master Mix on an ABI 7500 system. Relative expression levels were calculated using the ΔΔCt method with β‑actin as the internal control. Primer sequences were: 1190005I06Rik: 5′‑TGGTGTCCCTCACGGCCG‑3′ (forward), 5′‑TCAGACAGGAGGGCTTCAGC‑3′ (reverse); β‑actin: 5′‑TTACCAACTGGGACGACATG‑3′ (forward), 5′‑AGGGACAGCACAGCCTGGAT‑3′ (reverse). Samples were run in triplicate and averaged.

### Histological analysis

Eyes were enucleated and fixed in 4% paraformaldehyde (PFA) in PBS for 2 h at room temperature, then cryoprotected through a sucrose gradient (10%, 20%, 30% sucrose in PBS) and embedded in paraffin. Sections of eyes were cut at 8 μm thickness along the vertical meridian through the optic nerve. Sections were mounted on glass slides and used for morphological staining. Standard hematoxylin and eosin (H&E) staining was performed on retinal sections: slides were rehydrated, stained with hematoxylin solution (5 mins) and eosin (1 min), then dehydrated and coverslipped. Stained sections were examined by light microscopy (Zeiss Axio Imager). Retinal layer thicknesses (ONL, INL, total thickness) were measured using ImageJ. Three mice per genotype were analysed.

### Electroretinography (ERG)

Mice were dark‑adapted overnight and anaesthetised with pentobarbital sodium (1%; 0.1 mL/20 g). The pupils were dilated with 1% tropicamide ophthalmic drops, and a transparent viscoelastic gel (carbomer-based eye gel) was applied to keep the cornea moist and to ensure good contact with the recording electrode. For ERG recordings, a gold-ring electrode was positioned on the corneal surface of one eye as the active electrode, a reference electrode was positioned subcutaneously in the cheek or forehead area, and a ground electrode was inserted into the tail. Scotopic and photopic ERGs were recorded using an OPTO‑III electrophysiology system (Optoprobe) following standard protocols. The a- and b-wave amplitudes were measured. Recordings were performed in a blinded manner on at least three mice per genotype.

### Behavioral assays

All behavioral experiments were carried out using six control–experimental pairs during the light phase of the cycle in a quiet testing room, and mice were acclimated to the testing environment at least 4 h before each assay. Experimenters were blinded to genotype during behavioral testing and analysis. The data were analyzed by the tracking software (Any-maze).

Light/Dark Box Test: The apparatus consisted of a standard two-chamber box (one compartment brightly illuminated (~ 500 lux, white walls) and the other compartment dark (~ 0 lux, black walls), connected by a small doorway). Each mouse was placed in the dark compartment at the start of the 10-minute trial and allowed to freely explore both chambers. An overhead camera recorded the animal’s movements.

Visual Cliff Test: The visual cliff apparatus consisted of a clear glass platform with a patterned floor directly underneath one half (“shallow side”) and the same pattern dropped ~ 60 cm below the glass on the other half (“deep side”), creating the illusion of a cliff. Each mouse was placed on the central bridge of the platform and observed for 5 min. We recorded whether the mouse stepped off the center onto the shallow (safe) side or the deep (cliff) side, and the latency to the first choice.

## Supplementary Information

Below is the link to the electronic supplementary material.

Supplementary Material 1

## Data Availability

The raw sequence data reported in this paper have been deposited in the Genome Sequence Archive in National Genomics Data Center [28], China National Center for Bioinformation / Beijing Institute of Genomics, Chinese Academy of Sciences (GSA: CRA034022) that are publicly accessible at https://ngdc.cncb.ac.cn/gsa [29].
