# Supplementary material for: Systematic discovery of retina-enriched Rik genes identifies 1190005I06Rik as a novel modulator of visual signalling
Source: J Transl Med. 2026 Jan 30;24:296. doi: 10.1186/s12967-026-07769-z (PMC12930979; doi:10.1186/s12967-026-07769-z)
Supplement: Supplementary file 1 — Supplementary Material 1 [file 12967_2026_7769_MOESM1_ESM.docx]

Supplementary Materials for

Systematic discovery of retina-enriched Rik genes identifies 1190005I06Rik as a novel modulator of visual signalling

Yu-Tong Liu, Qing Li, Xinghai Yu, Zi-Wu Wang*, and Jun-Yan Kang *

*Correspondence to: kangjunyan@outlook.com (J.-Y.K.); wangziwu1@126.com (W.-Z.W.)

**This file includes:**

Figs. S1 to S5

**
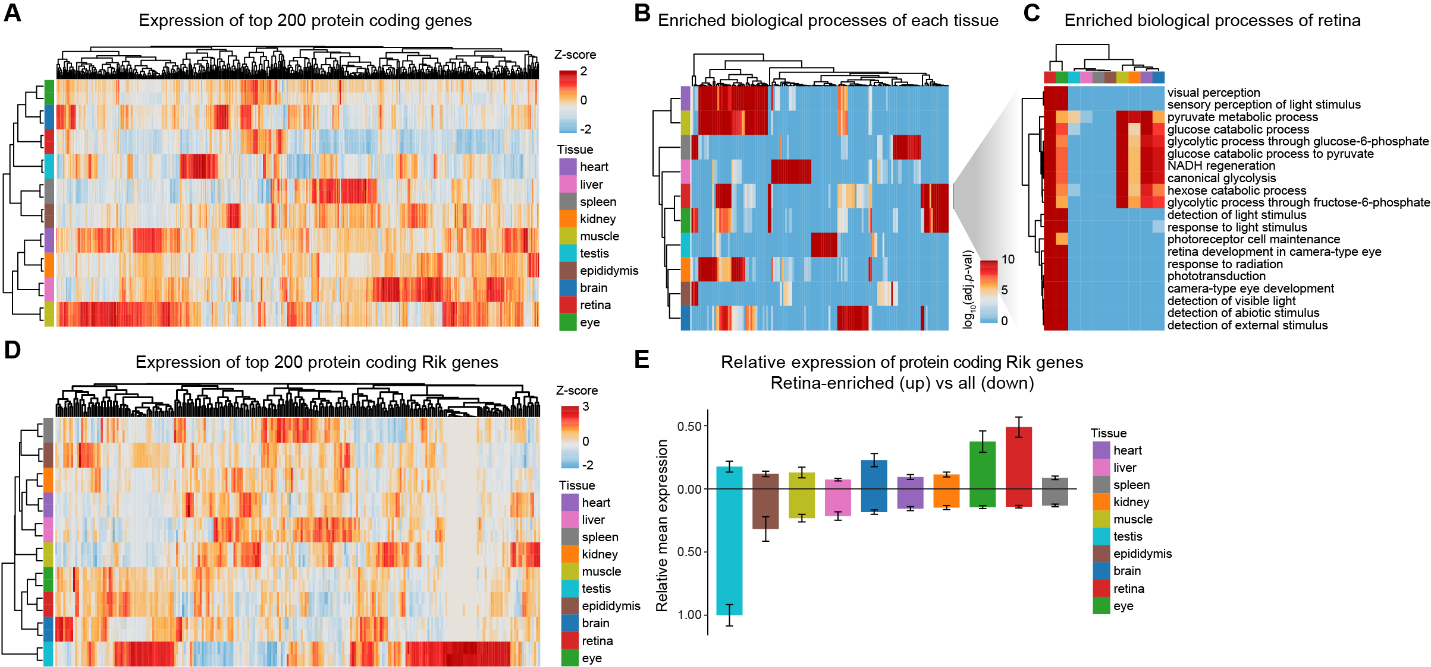
**

**Fig. S1. Transcriptomic features of all** **protein-coding genes and protein-coding Rik genes.** (**A**) Tissue-level expression heatmap of top 200 protein-coding genes*.* Z-score normalized log_2_FPKM values are shown across 10 tissues. Genes were ranked by mean expression, and rows clustered to reveal dominant tissue-specific modules. (**B**) Gene ontology (GO) enrichment of tissue-specific protein-coding genes*.* The top 200 protein-coding genes per tissue were used for GO enrichment (Biological Process category). Heatmap displays -log_10_ adjusted *p*-values for the top enriched terms across tissues. (**C**) GO enrichment analysis for retina-enriched coding genes. Subset of retina-enriched protein-coding genes were analyzed separately. Heatmap shows top GO terms exclusively enriched in retina, with biological relevance to light stimulus, energy production, and phototransduction. (**D**) Tissue-level expression heatmap of top 200 Rik genes. Z-score normalized log_2_FPKM values are shown across 10 tissues. Genes were ranked by average expression. Hierarchical clustering reveals shared and tissue-specific expression modules. (**E**) Relative expression of all Rik genes versus retina-enriched Rik genes. Diverging bar plot shows tissue-normalized mean ± SEM expression. All Rik genes are plotted downward, while the retina-enriched Rik genes are plotted upward.

**
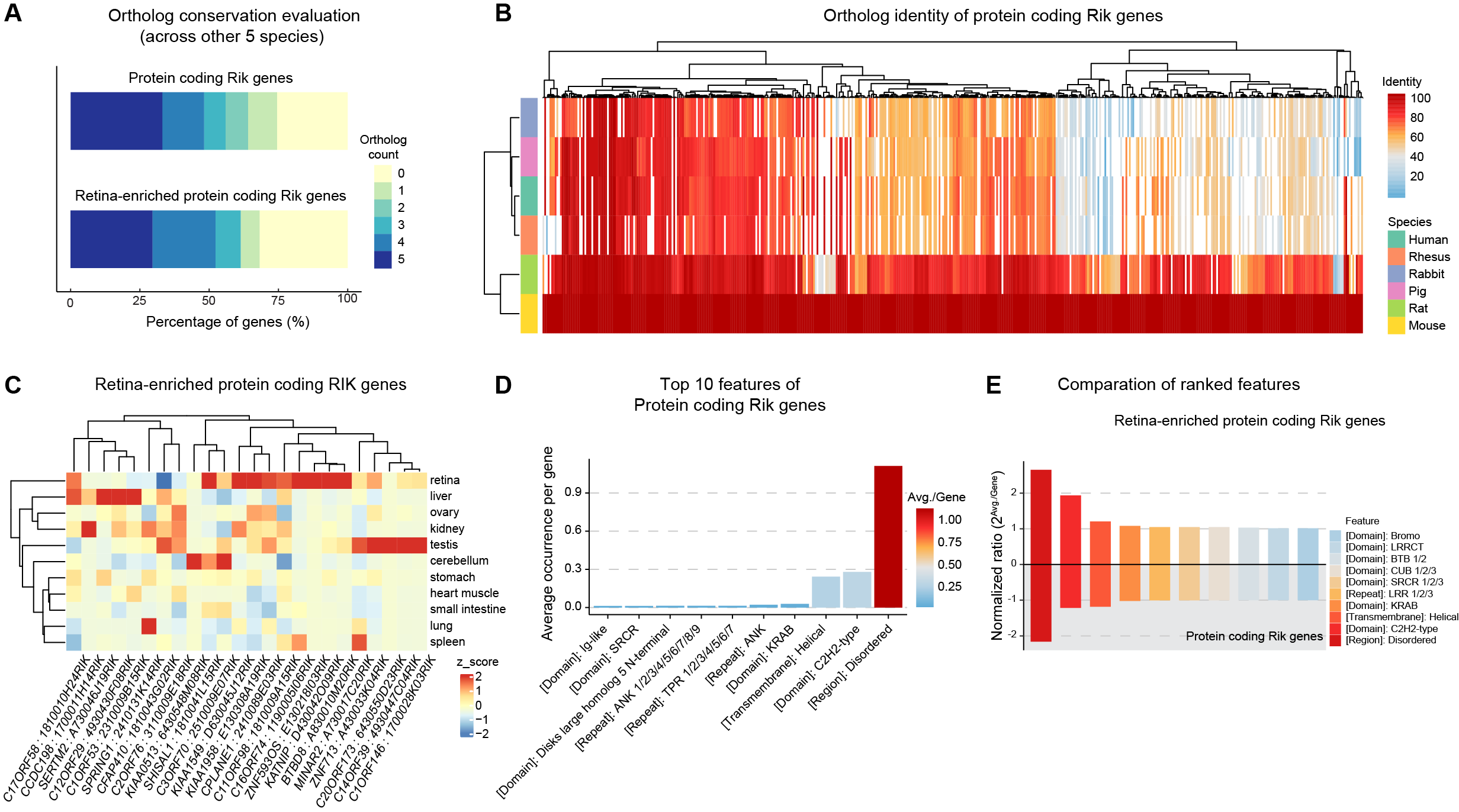
**

**Fig. S2. Ortholog breadth and protein feature analysis of Rik genes.** (**A**) Bar chart classifies each gene by the number of orthologs found across five non-mouse species. Retina-enriched Rik genes have a less tendency for cross-species conservation than the remaining Rik genes. (**B**) Extension of Fig. 2B showing identity scores for all protein-coding Rik genes. (**C**) Expression of human orthologues of mouse retina-enriched Rik genes (n = 24) across multiple human tissues. (**D**) Bar plot of the most common structural features across all protein-coding Riks, averaged per genes. (**E**) Diverging bar plot displaying differential enrichment of UniProt features between retina-enriched (top) and all protein-coding Riks (bottom).

**
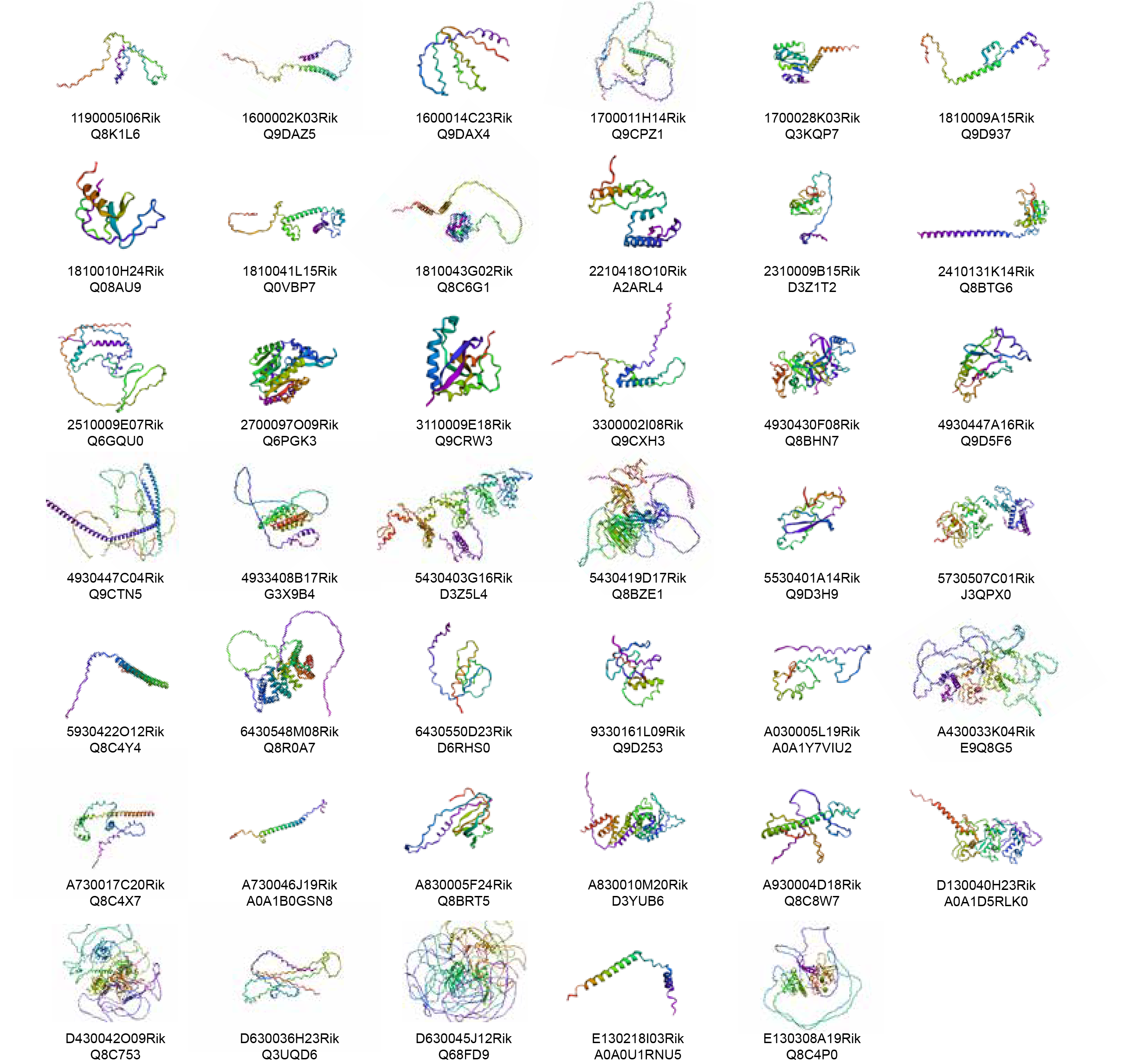
**

**Fig. S3. AlphaFold-based structural modeling of retina-enriched RIK proteins.** Predicted 3D structures of retina-enriched Rik proteins rendered via AlphaFold with color-coded cartoon representations. Labels include gene symbol and UniProt accession. These predictions suggest diverse structural patterns among retina-enriched RIK proteins despite short sequences and low conservation.


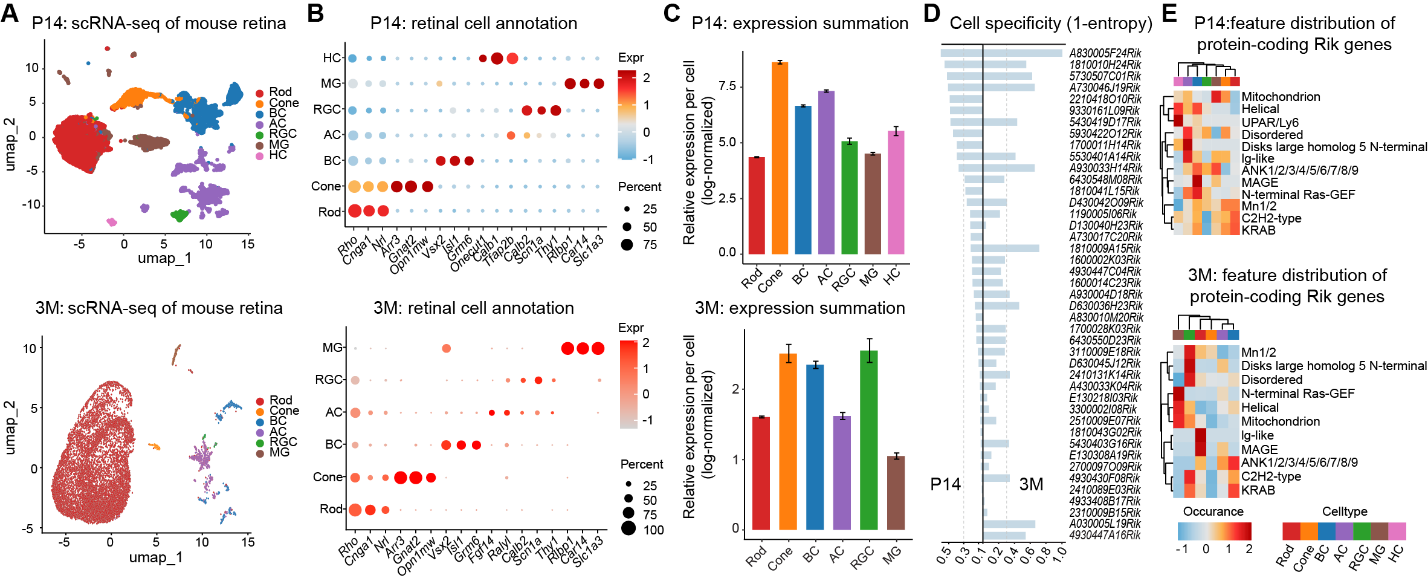


**Fig. S4. Single-cell analysis of retina-enriched Rik genes.** (**A**) Uniform Manifold Approximation and Projection (UMAP) plot of P14 (top) and 3M (bottom) mouse retinal cells, colored by manually curated cell type annotations. A total of 7 major retinal cell types from P14 mice were defined, including photoreceptors (27,957 Rods, 2,841 Cones), bipolar cells (7,860 BCs), horizontal cells (295 HCs), amacrine cells (5,721 ACs), retinal ganglion cells (588 RGCs), and Müller glia (2,763 MGs). A total of 6 major retinal cell types from 3M mice were defined, including photoreceptors (7,969 Rods, 81 Cones), 456 BCs, 534 ACs, 54 RGCs, and 141 MGs. (**B**) Dot plot showing the expression of representative marker genes across defined P14 (top) and 3M (bottom) retinal cell types. Dot size represents the proportion of cells expressing the gene within each cell type; color intensity reflects the average expression (log-normalized). Marker genes confirm canonical identities of different retinal cell types. (**C**) Boxplot summarizing the total expression levels (sum of log-normalized expression) of retina-enriched protein-coding Rik genes across each retinal cell type in P14 (top) and 3M (bottom) mice. (**D**) Bar plot ranking major retinal cell types by the average specificity score of retina-enriched Rik genes in P14 (left) and 3M (right) mice, ordered from highest to lowest specificity in P14. Specificity was computed as an entropy-based metric across all cell types (lower entropy = higher specificity). (**E**) Heatmap showing the relative feature-cell type enrichment of protein-coding RIK proteins across each retinal cell type in P14 (top) and 3M (bottom) mice. For each structural protein feature, the average expression across cell types was first computed and then normalized per gene (i.e., row-wise Z-score). Significance assessed by Wilcoxon rank-sum test with BH correction.

**
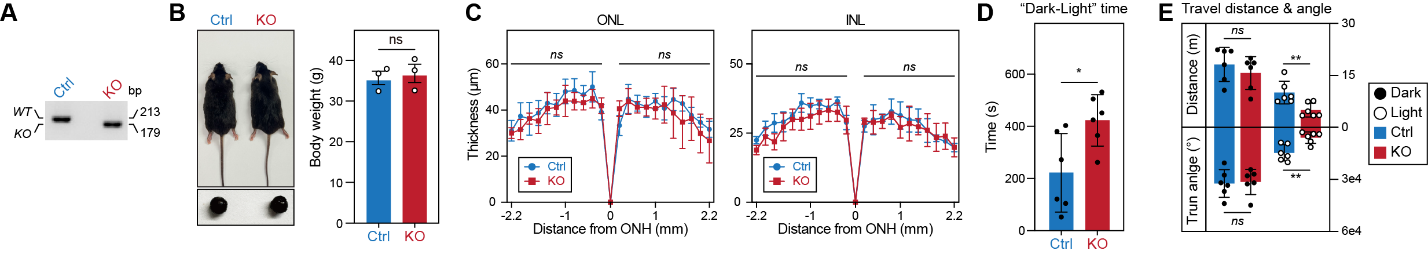
**

**Fig. S5. Tissue and developmental expression of *1190005I06Rik*.** (**A**) Genotyping of control and KO mice. Agarose gel electrophoresis of genomic DNA PCR products distinguishes the WT and KO alleles, as labeled. (**B**) Left, representative images of adult control and KO mice (top) and eyes (bottom). Right, quantification of body weight, plotted as mean ± SEM (n = 3 per genotype). (**C**) Spider graphs showing the outer nuclear layer (ONL, left) and inner nuclear layer (INL, right) thicknesses of control and KO retinas. Data represent mean ± SD (n = 3). **(D)** Quantification of the difference (time spent in dark - light) shown in the upper panel of Fig. 4I. Bar plots represent mean ± SD. **(E)** Quantification of total distance travelled (top) and absolute turn angle (bottom) in each zone. Significant differences (Student’s *t*-test) are marked with asterisks: **p* < 0.05, ** *p* < 0.01
